# Comprehensive Analysis of Codon Usage in *Quercus* Chloroplast Genome and Focus on *psbA* Gene

**DOI:** 10.3390/genes13112156

**Published:** 2022-11-18

**Authors:** Sheng-Lin Shi, Yan-Qun Liu, Run-Xi Xia, Li Qin

**Affiliations:** College of Bioscience and Biotechnology, Shenyang Agricultural University, Shenyang 110866, China

**Keywords:** *Quercus* species, chloroplast genome, codon usage, context-dependent mutation, translational selection, *psbA* gene, atypical codons

## Abstract

*Quercus* (oak) is an important economic and ecological tree species in the world, and it is the necessary feed for oak silkworm feeding. Chloroplasts play an important role in green plants but the codon usage of oak chloroplast genomes is not fully studied. We examined the codon usage of the oak chloroplast genomes in detail to facilitate the understanding of their biology and evolution. We downloaded all the protein coding genes of 26 non-redundant chloroplast reference genomes, removed short ones and those containing internal stop codons, and finally retained 50 genes shared by all genomes for comparative analyses. The base composition, codon bias, and codon preference are not significantly different between genomes but are significantly different among genes within these genomes. Oak chloroplast genomes prefer T/A-ending codons and avoid C/G-ending codons, and the *psbA* gene has the same preference except for the codons encoding amino acid Phe. Complex factors such as context-dependent mutations are the major factors affecting codon usage in these genomes, while selection plays an important role on the *psbA* gene. Our study provided an important understanding of codon usage in the oak chloroplast genomes and found that the *psbA* gene has nearly the same codon usage preference as other genes in the oak chloroplasts.

## 1. Introduction

Oak tree is the common name for species of genus *Quercus* in the *Fagaceae* family. Chinese oak silkworm (*Antheraea pernyi*) is one of the most famous wild silkworms, feeding on oak trees, hence the name [1]. The oak silkworm that feeds on artificial food cannot go through the complete larval stage, and the oak silkworm breeding industry is highly dependent on the resources and quality of oak trees. In addition, oak is the most important economic tree species in wood production and has important ecological roles [2,3]. So far, more than 500 species of oaks have been reported worldwide, of which 67 species are native to China [4,5]. However, in China, only less than 10 of these species are suitable and used for Chinese oak silkworm feeding, among which Liaotung oak (*Quercus wutaishanica*), Mongolian oak (*Q. mongolica*), Sawtooth oak (*Q. acutissima*), and Daimyo oak (*Q. dentata*) account for 95% of the total feed [6].

Chloroplasts play an important role in green plant photosynthesis, plant physiology and development, and serve as metabolic centers [7,8]. Chloroplasts also play a key role in plant immunity as integrators of non-self signals [9,10], and the chloroplast protein quality control system (such as the heat shock proteins) maintains the physiological functions of chloroplasts, so chloroplasts play a special role in heat stress response [11,12]. Land plant chloroplast genomes have a quadripartite circular structure comprising two copies of an inverted repeat region separated by a large single-copy region and a small single-copy region. Independent of nuclear genome, chloroplast contains a genome of approximately 115–165 kb in size, encoding approximately 130 genes, including approximately 90 proteins and 30 tRNAs [4,7]. Next generation sequencing (NGS) technology has increased the number of chloroplast sequences in the National Center for Biotechnology Information (NCBI) genome database [13]. These chloroplast genome sequences contribute to our understanding of plant biology, evolution, and diversity [14,15]. On the other hand, chloroplast genome engineering has brought many biotechnological applications, including improving plant resistance to biotic and abiotic stresses, producing high-value proteins, and enhancing biomass and nutrition [7]. Therefore, further in-depth understanding of the chloroplast structure, gene function, codon usage, etc., is required.

Synonymous codons are used uneven (termed codon bias) in all genes, all organisms, and are important for studies of evolutionary adaptation and biotechnology applications [16,17]. In recent years, researchers investigated the codon usage of several chloroplast genomes and found certain common patterns. Codon usage in green plant chloroplast genomes is relatively consistent across species, favors codons ending in base A or T, and is under pressure from both natural selection and mutational bias [18,19,20]. A study of angiosperm chloroplasts revealed that context-dependent mutations explain the codon usage bias of most angiosperm chloroplast genes, whereas codon usage of the highly expressed *psbA* gene is controlled by selection [21]. Oak chloroplast genome is about 160–161 kb in size, with similar gene organization and GC content among species [22,23]. However, the codon usage of oak chloroplast genomes is not reported in detail, except a simple codon usage table is often provided along with the genome reports [24,25]. The protein-coding sequences also prefer codons ending in A or T [26,27], as is the case in other chloroplast genomes [21,28].

Although the codon usage in green plant chloroplasts is similar between species, there are differences between genes within the genomes [29], and there are also two opinions on the role of selection in codon usage: play a major role or not play a major role [21]. Oaks are economically and ecologically important species worldwide, but the inter- and intra-genome information about the codon usage of their chloroplast genomes remains unclear. We performed a comprehensive codon usage comparison using 26 oak chloroplast reference genomes and focused on the codon usage of the *psbA* gene. Our results showed that the codon usage is similar across oak chloroplast genomes, but differs across genes within the genome, and context-dependent mutation is the main force affecting the codon usage. The *psbA* gene in oak chloroplast genomes is not as atypical as the *psbA* gene in chloroplast genomes of most angiosperms in that it only has one 2-fold degenerate amino acid (Phe) that prefers the C-ending codon.

## 2. Materials and Methods

### 2.1. Sequence Data

There were 28 *Quercus* chloroplast genomes available in the GenBank genome database (https://www.ncbi.nlm.nih.gov/genome/ accessed on 15 October 2021). The chloroplast genome sequence of *Q. wutaishanica* (NC_043857.1) is identical to that of *Q. fenchengensis* (NC_048513.1) though only one copy of gene *rps12* was annotated in *Q. wutaishanica*. To reduce redundancy, the chloroplast genome of *Q. fenchengensis* was excluded. The chloroplast genome of *Q. mongolica* subsp. *crispula* (NC_049877.1) was excluded too because it is a subspecies and the chloroplast genome of *Q. mongolica* (NC_043858.1) was already included in the data. Therefore, we selected the remaining 26 non-redundant chloroplast reference genomes for further study (Table 1). Among the 26 chloroplast genomes, the chloroplast genomes of *Q. dentata* (NC_039725.1), *Q. wutaishanica* (NC_043857.1), and *Q. mongolica* (NC_043858.1) were completed and submitted by our laboratory.

We extracted all protein coding genes from the 26 genomes (including the missed *rps12* gene in the *Q. wutaishanica* genome), removed codons containing ambiguous bases in all genes, and removed genes lacking proper stop codons and genes encoding less than 100 amino acids. Blast analysis showed that *psi* gene, which is only present in the *Q. rubra* chloroplast genome (NC_020152.1), is highly matched with the *psbB* gene in other chloroplast genomes, so we re-annotated the *psi* gene as *psbB* gene for subsequent analysis. The *ndhJ* gene in the chloroplast genome of *Q. coccinea* (NC_047481.1) is misannotated at genomic positions 61,506–62,009, and the misannotated *ndhJ* gene is contained within the *rbcL* gene (at genomic positions 60,582–62,009), so we re-annotated this *ndhJ* gene (at genomic positions 54,531–55,007) for subsequent analysis.

After these data filtering and correction process, the numbers of genes remaining in these genomes varied from 55 to 58. We further excluded the second copy of the genes (*ndhB*, *rpl2*, *rps12*, *rps7*, *ycf2*) that have two copies in each genome. To facilitate inter- and intra-genome comparisons, we finally included only the 50 genes that were shared by all genomes in our research (*accD*, *atpA*, *atpB*, *atpE*, *atpF*, *atpI*, *ccsA*, *cemA*, *clpP*, *matK*, *ndhA*, *ndhB*, *ndhC*, *ndhD*, *ndhF*, *ndhG*, *ndhH*, *ndhI*, *ndhJ*, *ndhK*, *petA*, *petB*, *petD*, *psaA*, *psaB*, *psbA*, *psbB*, *psbC*, *psbD*, *rbcL*, *rpl14*, *rpl16*, *rpl2*, *rpl20*, *rpoA*, *rpoB*, *rpoC1*, *rpoC2*, *rps11*, *rps12*, *rps18*, *rps2*, *rps3*, *rps4*, *rps7*, *rps8*, *ycf1*, *ycf2*, *ycf3*, *ycf4*).

### 2.2. Nucleotide Composition and Codon Bias Indices

The nucleotide composition of guanine plus cytosine (G + C) at the first, second, and third positions of synonymous codons (termed GC1s, GC2s, and GC3s, respectively) were counted for each gene. The effective number of codons (*N*_c_), first introduced by Frank Wright, assigns a number to a gene to quantify the degree of codon usage bias [48]. Wright’s *N*_c_ would take a minimum value of 20 for a gene with extreme codon usage bias (each amino acid used only one of the synonymous codons) and would take a maximum value of 61 for a gene without codon usage bias (all amino acids used all possible codons equally). Wright’s *N*_c_ does not require a reference gene and is therefore widely used to characterize codon usage bias. However, the value of Wright’s *N*_c_ may be much larger than the number of sense codons. Later, Sun, et al. [49] developed a new *N*_c_ based on Wright’s *N*_c_, which is superior to Wright’s *N*_c_. The new *N*_c_ breaks the 6-fold codon families into 2-fold and 4-fold families and thus its value would range from the number of codon families (minimum value) to the number of sense codons (maximum value). Both Wright’s *N*_c_ and Sun’s new *N*_c_ are calculated from the number of codons of the gene under study and are estimators (estimated *N*_c_) of the “true” values of *N*_c_.

### 2.3. Relative Synonymous Codon Usage

Relative synonymous codon usage (RSCU) is defined as the number of synonymous codons observed in an amino acid divided by the expected number of the codons if all synonymous codons for this amino acid are equally used [50]. An RSCU value equal to one means no codon usage bias, while an RSCU value greater than one represents a positive codon usage bias, and vice versa. A cosine similarity index is introduced to measure the similarity of codon usage between two coding sequences based on RSCU values [51]. Furthermore, codons with RSCU values smaller than 0.6 are considered under-represented, while codons with RSCU values greater than 1.6 are considered over-represented [52,53].

### 2.4. Estimated N_c_ versus Expected N_c_ Plot and P_12_ versus P_3_ Plot

There is a close relationship between codon usage bias and GC3s, and the variation in GC3s will complicate the interpretation of the estimated *N*_c_ value for a given gene [48]. When there is no translational selection, the estimated *N*_c_ can simply be approximated from GC3s and this time the estimated *N*_c_ is the expected *N*_c_. The estimated *N*_c_ values versus the expected *N*_c_ values were plotted against GC3s content to examine the influence of nucleotide composition on codon usage as described by Frank Wright [48]. A gene restricted by GC composition will possess an estimated *N*_c_ equal to or nearly equal to the expected *N*_c_, while a gene under translational selection will have an estimated *N*_c_ much smaller than the expected *N*_c_ [48].

The *P*_12_ versus *P*_3_ plot is based on the directional mutation theory and is used to estimate the extent of directional mutation pressure against selective constraints [54,55]. In the analysis, *P*_1_, *P*_2_, and *P*_3_ are the observed GC contents of the first, second, and third codon positions of individual genes, respectively. *P*_12_ is the average of *P*_1_ and *P*_2_ and six codons (ATG, TGG, ATA, TAA, TAG, or TGA) are excluded from the calculation. When plotting *P*_12_ against *P*_3_, the regression coefficient is usually used as the mutation-selection equilibrium coefficient.

### 2.5. Tests for Context-Dependent Mutation

The three 6-fold degenerate amino acids (Arg, Leu, and Ser) can be divided into three 2-fold degenerate families (Arg2, Leu2, and Ser2) and three 4-fold degenerate families (Arg4, Leu4, and Ser4), so there will be eight 4-fold degenerate families. On the condition that mutation is an independent single-site event, the nucleotide frequencies of the third codon position in the 4-fold degenerate families will not be affected by the second and/or the first codon positions. According to the method employed in the codon usage investigation of mitochondria [56,57], we tested the independence of the third codon position in the eight 4-fold degenerate families. We used six datasets in this analysis: Leu4/Pro/Arg4; Val/Ala/Gly; (Leu4 + Val)/(Ser4 + Pro + Thr + Ala)/(Arg4 + Gly); Leu4/Val; Arg4/Gly; Ser4/Pro/Thr/Ala. The Chi-square test of independence was conducted for each dataset.

### 2.6. Software and Calculation

We used Perl scripts (Code S1) to calculate the GC1s, GC2s, and GC3s, as well as *P*_12_ and *P*_3_. Sun’s (estimated) *N*_c_ and the expected *N*_c_ were calculated using software DAMBE 7.3.2 [58]. The codon number and RSCU value were also calculated using perl script (Code S2). Statistical analysis and similarity index calculations were performed using software SPSS 16.0. The heat map was drawn using software HemI 1.0, and the row/column data of the heat map were clustered by the average linkage method using the Euclidean distance [59].

## 3. Results

### 3.1. Base Composition of Synonymous Codons

**Figure 1:**
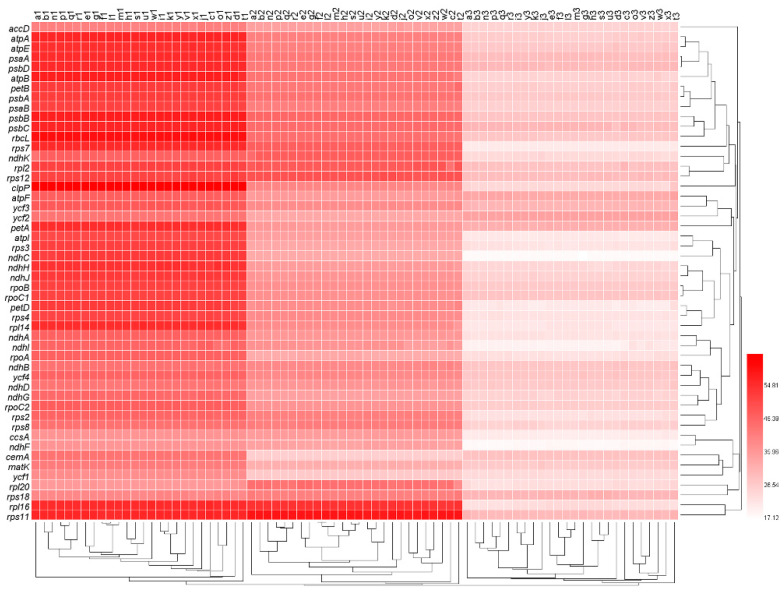
The nucleotide composition at the first, second, and third codon positions of all genes. The numbers next to the legend indicate the percentage of GC content. Lowercase letters from a to z at the top of the heat map represent the data sets of oak trees *Q. rubra*, *Q. aliena*, *Q. spinose*, *Q. aquifolioides*, *Q. baronii*, *Q. variabilis*, *Q. dolicholepis*, *Q. tarokoensis*, *Q. glauca*, *Q. tungmaiensis*, *Q. sichourensis*, *Q. chenii*, *Q. acutissima*, *Q. dentate*, *Q. obovatifolia*, *Q. wutaishanica*, *Q. mongolica*, *Q. robur*, *Q. bawanglingensis*, *Q. coccinea*, *Q. phillyraeoides*, *Q. gilva*, *Q. pannosa*, *Q. virginiana*, *Q. acuta,* and *Q. chungii*, respectively. The numbers 1, 2, and 3 after the lowercase letters represent the 1st, 2nd, and 3rd positions of the synonymous codons, respectively.

We measured the GC contents at the first, second, and third codon positions (termed GC1s, GC2s, and GC3s, respectively) of the 50 genes in each of the 26 genomes (Table S1). In the clustered heat map of GC contents (Figure 1), there were large differences between GC1s, GC2s, and GC3s of each genome, and GC1s, GC2s, and GC3s formed three larger clades, respectively. Moreover, GC1s, GC2s, and GC3s all showed obvious differences among genes, but smaller differences among genomes. Genes *psbA*, *petB,* and *psaB* first formed a smaller clade, and then together with the clade of genes *atpA*, *atpE*, *psaA*, *psbD,* and *atpB*, and the clade of genes *psbB*, *psbC,* and *rbcL* formed a larger clade (Figure 1), indicating that the GC content of *psbA* was closely related to these genes.

**Figure 2:**
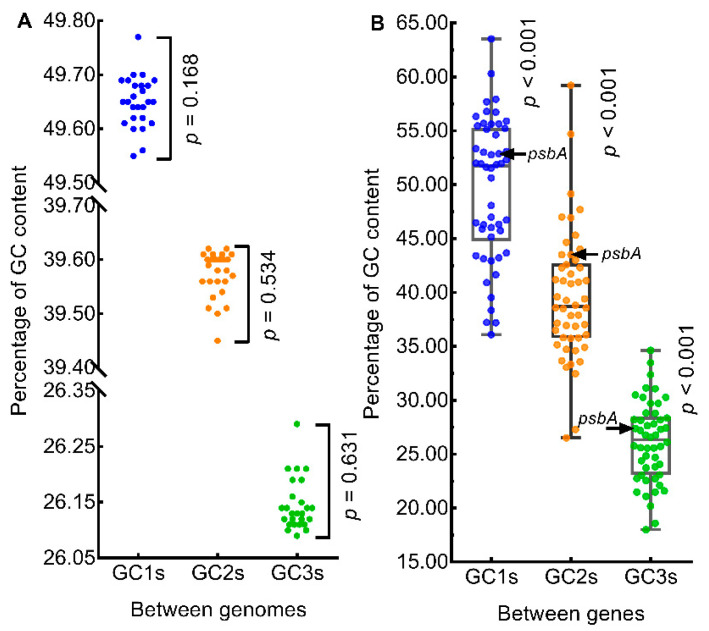
Comparison of nucleotide contents. (**A**) GC content of genomes. (**B**) GC content of genes. The color circles in panels (**A**) and (**B**) represent the mean GC contents of the genomes (*n* = 26) and the mean GC contents of genes (*n* = 50), respectively.

Between genomes (Figure 2A), GC1s ranged from 49.55 (SD = 6.47, *n* = 50, *Q. coccinea*) to 49.77 (SD = 6.55, *n* = 50, *Q. aquifolioides*), GC2s ranged from 39.45 (SD = 6.06, *n* = 50, *Q. coccinea*) to 39.62 (SD = 6.03, *n* = 50, *Q. aliena*), GC3s ranged from 26.09 (SD = 3.76, *n* = 50, *Q. aliena*) to 26.29 (SD = 3.66, *n* = 50, *Q. coccinea*). GC1s, GC2s, and GC3s were not significantly different between genomes (*n* = 26; *p* = 0.168, *p* = 0.534, *p* = 0.631, respectively; Repeated Measures ANOVA). Within genomes (Figure 2B), GC1s ranged from 36.10 (SD = 0.41, *n* = 26, *rpl20*) to 63.53 (SD = 0.25, *n* = 26, *clpP*), GC2s ranged from 26.52 (SD = 0.07, *n* = 26, *cemA*) to 59.23 (SD = 0.32, *n* = 26, *rps11*), GC3s ranged from 17.99 (SD = 0.18, *n* = 26, *ndhC*) to 34.64 (SD = 0.05, *n* = 26, *ycf2*). GC1s, GC2s, and GC3s were significantly different between genes within genomes (*n* = 50, *p* < 0.001 in all three cases, Repeated Measures ANOVA). GC1s, GC2s, and GC3s of *psbA* gene were 52.87 (SD = 0.01, *n* = 26), 43.50 (SD = 0.06, *n* = 26) and 27.38 (SD = 0.26, *n* = 26), respectively, and the percentile scores were 62, 78, and 60, respectively (Figure 2B); this indicated that the GC content of the *psbA* gene was within the normal range for oak chloroplast genomes.

### 3.2. Degree of Codon Usage Bias

**Figure 3:**
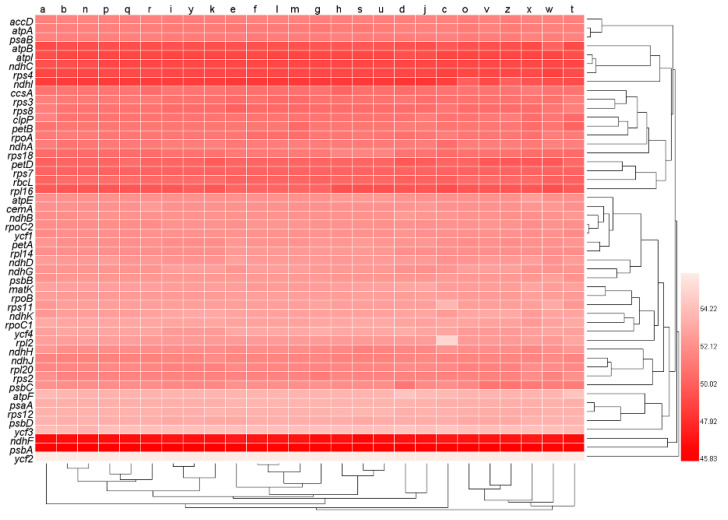
Codon usage bias measured by effective number of codons (*N*_c_) of all genes. The numbers next to the legend indicate values of *N*_c_. Lowercase letters from a to z at the top of the heat map represent the data sets of oak trees *Q. rubra*, *Q. aliena*, *Q. spinose*, *Q. aquifolioides*, *Q. baronii*, *Q. variabilis*, *Q. dolicholepis*, *Q. tarokoensis*, *Q. glauca*, *Q. tungmaiensis*, *Q. sichourensis*, *Q. chenii*, *Q. acutissima*, *Q. dentate*, *Q. obovatifolia*, *Q. wutaishanica*, *Q. mongolica*, *Q. robur*, *Q. bawanglingensis*, *Q. coccinea*, *Q. phillyraeoides*, *Q. gilva*, *Q. pannosa*, *Q. virginiana*, *Q. acuta,* and *Q. chungii*, respectively.

We employed *N*_c_ to differentiate the degree of codon usage bias in all the 50 genes × 26 genomes (Table S1). In the clustered heat map (Figure 3), the *N*_c_ values were similar among genomes, but varied widely among genes, consistent with the heat map pattern of GC content in Figure 1. Genes *psbA* and *ndhF* formed a clade that had the lowest *N*_c_ value, genes *atpB*, *atpI*, *ndhC*, *rps4,* and *ndhI* formed a clade that had the second lowest *N*_c_ value, and genes *petD*, *rps7*, *rbcL,* and *rpl16* formed a clade that had the third lowest *N*_c_ value. On the other hand, the *ycf2* gene had the highest *N*_c_ value and formed a clade alone, and genes *atpF*, *psaA*, *rps12*, *psbD,* and *ycf3* formed a clade with the second highest *N*_c_ value.

**Figure 4:**
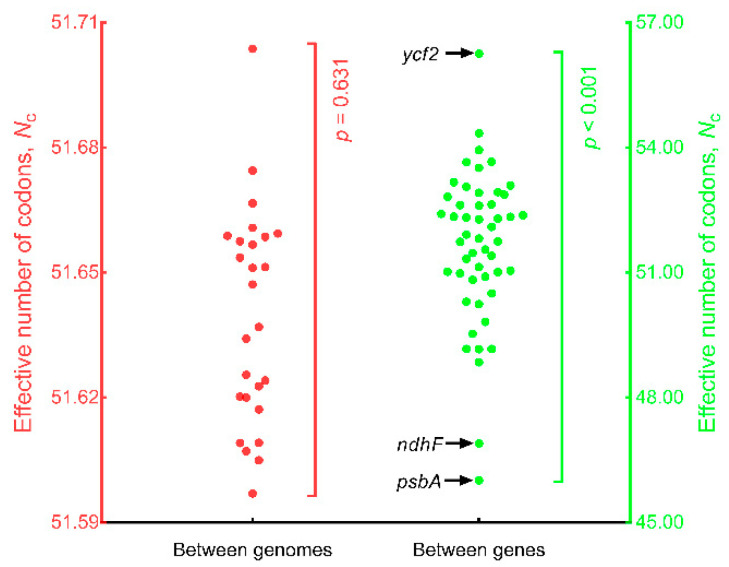
Comparison of effective number of codons (*N*_c_) between genomes and between genes. The 26 red solid circles represent the mean *N*_c_ values (*n* = 50) for each of the 26 genomes and are plotted on the left y-axis. The 50 green solid circles represent the mean *N*_c_ values (*n* = 26) for each of the 50 genes and are plotted on the right y-axis.

Between genomes (Figure 4), *N*_c_ ranged from 51.597 (SD = 1.851, *n* = 50, *Q. phillyraeoides*) to 51.704 (SD = 1.911, *n* = 50, *Q. spinosa*) and did not differ significantly (*n* = 26; *p* = 0.631; Repeated Measures ANOVA). Within genomes, *N*_c_ ranged from 46.009 (SD = 0.167, *n* = 26, *psbA*) to 56.250 (SD = 0.037, *n* = 26, *ycf2*) and differed significantly between genes (*n* = 50; *p* < 0.001; Repeated Measures ANOVA). These results indicated that *psbA* was the most biased gene in oak chloroplast genomes.

### 3.3. Pattern of Codon Usage Bias

We used RSCU to measure the codon usage preference of individual amino acids of all the 1300 genes (50 genes × 26 genomes) and colored all codons according to their RSCU values (Table S2). In all genomes, the genes *atpE* and *rps11* lacked amino acid Tyr; *ndhC* lacked amino acids Asn, Cys, and His; *rpl20* lacked amino acid Pro; *psbA* lacked amino acid Lys; and *rps18* and *rps7* lacked amino acid Cys. Gene *atpF* lacked amino acid Cys in 22 genomes. In Table S2, homologous genes across the genomes had the same or nearly the same RSCU values, and thus were colored the same or almost the same. By calculating the cosine similarity index, we found that (1) the similarity between genes within genomes was lower than the similarity between genomes, and (2) the similarity between genes within genomes was more diverse (larger range and standard deviation) than the similarity between genomes (Table S3). Therefore, in the subsequent analyses, we used the mean similarity index (*n* = 26, Table S4) and the mean RSCU values (*n* = 26, Table S5) of the homologous genes to infer the pattern of codon usage in the oak chloroplast genomes.

The mean similarity indices of all “gene versus gene” pairs ranged from 0.470 (*ndhC* versus *petD*) to 0.979 (*rpoB* versus *rpoC2*), with a range of 0.509, indicating that diversity existed in terms of codon usage pattern (Table S4). When a gene’s similarity indices (one versus all others) are summed to obtain a cumulate similarity index to represent the codon similarity of this gene, the *psaB* gene had the highest cumulate similarity index while the *ndhC* gene had the lowest cumulate similarity index (Table S4). The cumulate similarity index of the *psbA* gene ranked a percentile score of 20, higher than those of genes *ndhC*, *rpl16*, *rps18*, *atpF*, *rpl20*, *atpE*, *cemA*, *rps11*, *petD,* and *rps8*, indicating that the overall codon usage of the *psbA* gene was in the normal range too in terms of RSCU values (Table S4).

**Figure 5:**
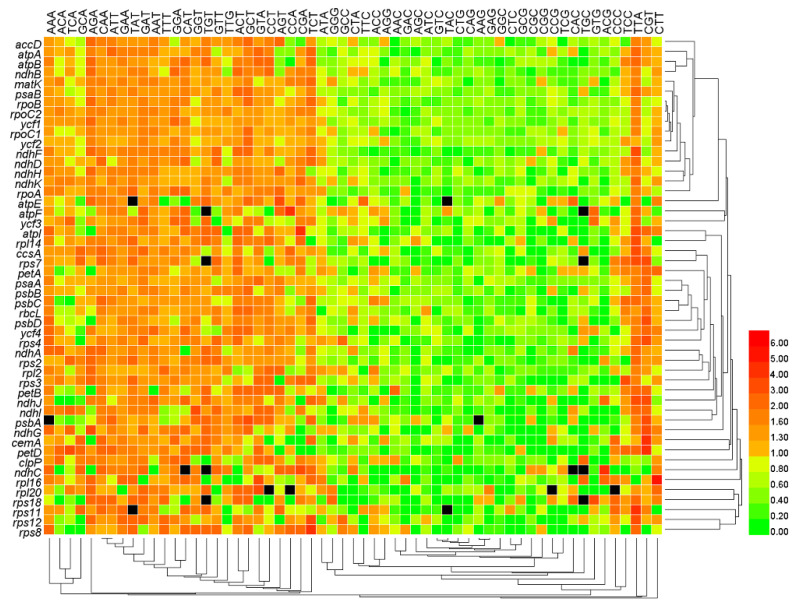
Heat map of codon usage preference based on mean RSCU values. Black cells in the heat map represent that the corresponding gene lacks the amino acid encoded by the corresponding codons. The numbers next to the legend represent RSCU values.

The mean RSCU showed discordance among genes, but no clear differences between different clustering clades of genes (Figure 5). The RSCU values of A/T-ending codons were relatively higher than C/G-ending codons. All codons were clustered into two large branches: one branch mainly consisting of codons ending in T/A and the other branch mainly consisting of codons ending in C/G (Figure 5). The clustering results of three codons were different: the codon CTA (encoding Leu) and codon ATA (encoding Ile) were clustered into the branch composed of codons ending in C/G, while the codon TTG (encoding Leu) was clustered into the branch composed of codons ending in T/A (Figure 5). To confirm these observations, we grouped all codons into four categories based on the third base of the codons: A-ending codons, C-ending codons, G-ending codons, and T-ending codons. Meanwhile, all codons were divided into six groups according to their RSCU values: (1) over-represented codons, RSCU > 1.6; (2) positive biased codons but not over-represented, RSCU > 1 and ≤ 1.6; (3) unbiased codons, RSCU = 1; (4) negative biased codons but not under-represented, RSCU < 1 and ≥ 0.6; (5) under-represented codons, RSCU < 0.6 and > 0; and (6) unused codons, RSCU = 0. After grouping, we found 81.56% of T-ending codons and 71.06% of A-ending codons were positive biased, while only 11.36% of C-ending codons and 13.27% of G-ending codons were positive biased (Table 2). The codon preference of the *psbA* gene was similar to that of the whole genome (50 genes) except that only less than half of A-ending codons were positive biased (Table 2), which indicated that the *psbA* gene also preferred A/T-ending codons.

### 3.4. The Third Base of Codon in Use

To detail the preference of codon usage in oak chloroplast genomes, the number of all codons for all 50 genes in all 26 genomes was calculated and summed by genome (Table S6). Each of the three 6-fold degenerate amino acids (Arg, Leu, and Ser) was divided into a 4-fold degenerate family and a 2-fold degenerate family. As a result, there were 12 2-fold degenerate families, one 3-fold degenerate family, and eight 4-fold degenerate families. Oak chloroplast genomes (in 26 out of 26 genomes) favored T- over C-ending codons in each of the seven 2-fold NNY type degenerate families (Asn, Asp, Cys, His, Phe, Tyr, Ser2), favored A- over G-ending codons in each of the five 2-fold NNR type degenerate families (Gln, Glu, Lys, Arg2, Leu2), and had a codon favor of ATT > ATA > ATC in the 3-fold Ile degenerate family. Oak chloroplast genomes (in 26 out of 26 genomes) also favored T- and A-ending codons over C- and G-ending in each of the eight 4-fold degenerate families (Ala, Gly, Pro, Thr, Val, Arg4, Leu4, Ser4). The *psbA* gene showed a similar codon preference to the oak genome with only few exceptions. In the 2-fold NNY type degenerate families, the *psbA* gene favored codon TTC over TTT (20:6) for Phe (in 26 out of 26 genomes), favored codon AGC over AGT (6:5) for Ser (in 5 out of 26 genomes), and favored neither AGC nor AGT (5:5) for Ser (in 21 out of 26 genomes). In the 3-fold Ile degenerate family, the *psbA* gene had a codon favor of ATT > ATC > ATA (20:6:4) (in 26 out of 26 genomes). In the three 4-fold degenerate families (Thr, Arg, and Ser), the *psbA* gene most favored T-ending codons, followed by C-ending codons, while the whole genomes favored T-ending codons, followed by A-ending codons (in 26 out of 26 genomes).

### 3.5. Factors Accounting for Codon Usage

Taking into account the results that the base composition, codon bias, and codon preference of homologous genes are very similar across genomes, we used the mean of homologous genes (*n* = 26) across genomes to represent each of the 50 genes in the chloroplast genomes. Correlation analysis showed that GC1s and GC2s were positive correlated (*ρ* = 0.505, *p* < 0.001, *n* = 50), but neither of the two was significantly correlated with GC3s (*ρ* = 0.146, *p* = 0.247, *n* = 50; *ρ* = 0.312, *p* = 0.084, *n* = 50, respectively). The correlation of *N*_c_ with GC3s was significant (*ρ* = 0.697, *p* < 0.001, *n* = 50), but not with GC1s and GC2s (*ρ* = –0.059, *p* = 0.683, *n* = 50; *ρ* = 0.028, *p* = 0.847, *n* = 50, respectively). These results suggested that GC3s affects the codon usage in *Quercus* chloroplast genome.

**Figure 6:**
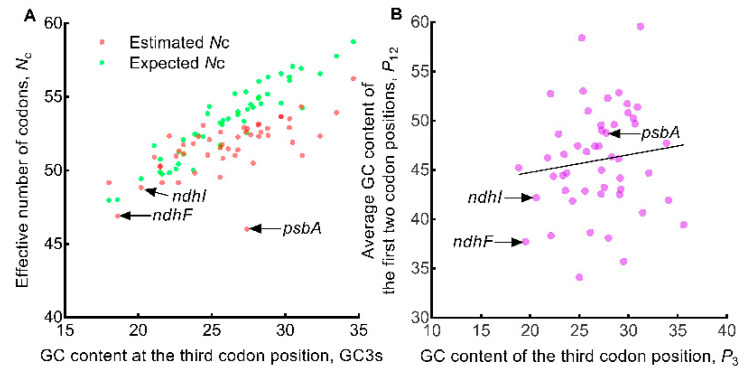
Exploring the influencing factors of codon usage in oak chloroplast genome. The solid color circles represent the 50 genes. (**A**) Estimated *N*_c_ (effective number of codons) versus expected *N*_c_ plot analysis. (**B**) *P*_12_ versus *P*_3_ plot analysis. The solid line represents the regression of *P*_12_ (average of *P*_1_ and *P*_2_) against *P*_3_, equation *y* = 0.1791*x* + 41.17, *R*^2^ = 0.0155, *p* = 0.389.

In the plotting of estimated *N*_c_ and expected *N*_c_ against GC3s (Figure 6A), the points that represent the estimated *N*_c_ and those that represent the expected *N*_c_ scattered similarly with the exception of the point of the *psbA* gene which was located far away from its expected point. For genes with relatively higher GC3s values, their estimated *N*_c_ values were also lower than their expected values. The *ndhF* and *ndhI* genes were clustered closely related to the *psbA* gene in Figure 3 and Figure 5, respectively. However, the points of the two genes, *ndhF* and *ndhI*, were both located next to their expected values. These results suggested that the factors affecting the *psbA* gene were different from all other genes, and that selection may be the main affecting factor for the *psbA* gene while mutation may be the main affecting factor for other genes. The *P*_12_ against *P*_3_ plotting only got an insignificant regression (*R*^2^ = 0.0155, *p* = 0.389) (Figure 6B) due to the narrow spread of *P*_3_ and the wide spread of *P*_12_, suggesting that the influencing factors (mutation or selection or both) exerted on the oak chloroplast genomes might be complex. In the *P*_12_ versus *P*_3_ plot, the *psbA* gene was located near the center, while the other two genes (*ndhF* and *ndhI*) that most associated with *psbA* were located near the edges of the plot.

We further tested the context-dependent mutation by analyzing the codon frequency in the eight 4-fold degenerate families. The result showed that the variation of codons’ third base correlated with both the second base and the first base in genomes (Table 3), that is, there existed context-dependent mutations. However, the variation of codon’s third base only partially correlated with the second base in the *psbA* gene, and independent of the first base. Since the six datasets of *psbA* gene are much smaller, the insignificant *p* values may be due to low statistical power. We tested for context-dependent mutation in two genes, *ndhA* and *psbD*, which are of similar size to the *psbA* gene, and in two other highly translated genes, *rbcL* and *psbC*, and found similar insignificant *p* values to the *psbA* gene (Table S7).

## 4. Discussion

Understanding the evolution of chloroplast genomes is as important as obtaining their sequences. Codon usage bias is a key aspect of genome evolution and is a secondary genetic code that guides protein production [60]. We performed a detailed analysis of codon usage in the *Quercus* chloroplast genomes. In terms of nucleotide composition at three codon positions (GC1s, GC2s, and GC3s), together with the codon bias index *N*_c_ and RSCU values, *Quercus* chloroplasts showed no significant differences between genomes but significant differences between genes within the genomes. Codon usage in chloroplast genomes of *Theaceae* species was found to be similar between species and prefer A/T ending codons [61], in accordance with the results of 16 *Fagaceae* chloroplast genomes [27] and the results of our investigation. We found that codon usage was consistent between oak chloroplast genomes, and that most A- and T-ending codons had relatively higher RSCU values and were therefore preferred for use.

Most researchers analyzed codon usage of the chloroplast genomes as a whole and therefore only provided the overall codon usage of the entire genomes [19,29]. Recently, a study grouped the genes within the chloroplast genomes into two major categories (photosynthesis-related genes and genetic system-related genes) according to their functions, and found obvious differences between the two categories [29]. We believe that it is best to analyze each gene in the chloroplast genomes individually, otherwise if the codon usage of these genes is inconsistent, the overall codon usage of a category/genome will mask the codon usage characteristics of these individual genes within the genomes. In our study, the 50 genes shared in all the 26 genomes were analyzed one by one, and significant differences were found among the 50 genes. A study found differential selection acted on the *atpF* gene between evergreen sclerophyllous oak (*Q. aquifolioides*) and deciduous species (*Q. rubra* and *Q. aliena*) by measuring the ratio of non-synonymous to synonymous [32]. However, in our analysis, the *atpF* genes of the three species does not differ significantly from other genes in codon usage.

The *psbA* gene in chloroplast genomes was found to favor codon NNC over NNT for 2-fold NNY type degenerate amino acids, and selection acts on this gene for high translation efficiency [62,63,64]. In oak chloroplast genomes, however, this codon usage preference was only present for the amino acid Phe, which prefers the codon TTC over TTT, although selection still acted on the oak’s *psbA* gene. The GC3s of oak’s *psbA* gene was comparable to that of other genes in the oak chloroplast genomes, while in *Marchantia polymorpha,* the GC3s of the *psbA* gene was significantly higher than other genes [62]. Oak’s *psbA* gene ranked in the 20th percentile as measured by codon usage similarity index, and with the normal GC content and almost normal codon preference, we considered that oak’s *psbA* gene was not as atypical as the *psbA* gene in other angiosperm chloroplasts [63].

Selection and mutation are two major forces that shape the codon usage in the chloroplast genome. For example, in the chloroplast genomes of species in the *Asteraceae* family [65] and *Euphorbiaceae* family [20], selection was reported to play a major role, while mutation plays a minor role in codon usage. On the contrary, in the chloroplast genomes of *Oncidium Gower Ramsey* [66] and *Coffea arabica* [67], mutation was identified to play a major role, while selection plays a minor role. In the present study, we found that context-dependent mutation plays an important role in shaping the codon usage of most genes in the *Quercus* chloroplast genomes, which is consistent with the case of most angiosperm chloroplasts [21]. However, mutation acts differently on different genes within the genome, and selective constraints play a role in codon usage too [68]. We believe that the factors affecting the codon usage of different genes in the chloroplast genomes are complex and diverse, and the codon usage of different genes respond differently to the influencing factors, so more complex models or methods are needed to obtain more comprehensive and realistic research results.

## 5. Conclusions

We analyzed the codon usage in oak chloroplast genomes using 50 genes that were shared by all the 26 non-redundant chloroplast reference genomes. The results showed that base composition and codon bias do not differ significantly between genomes, while there are significant differences between genes within the genomes. Oak chloroplast genomes prefer T/A-ending codons and avoid C/G-ending codons. Codon preference among oak chloroplast genomes is relatively consistent, while among genes within the chloroplast genomes, it is relatively diverse. Context-dependent mutation plays a more decisive role than selection in shaping the codon usage of the oak chloroplast genomes. In oak chloroplast genomes, *psbA* is the most biased gene, but has a similar codon preference to the entire genomes, although the Phe encoded by the *psbA* gene prefers codon TTC rather than TTT, unlike other genes in the genomes. The codon usage of oak’s *psbA* gene differs from that of the *psbA* gene in other angiosperm chloroplast genomes, which were found to be atypical in that they prefer the C-ending codons for NNY type 2-fold amino acids instead of the T-ending codons.

## Figures and Tables

**Table 1:** Chloroplast genomes and their associated information of 26 *Quercus* species.

Accession No.	Species [Ref.]	Letter Series	Genome Size (bp)	Genome GC%	Protein Coding Genes
NC_020152.1	*Q. rubra* [4]	a	161,304	36.80	89
NC_026790.1	*Q. aliena* [30]	b	160,921	36.88	82
NC_026907.1	*Q. spinosa* [31]	c	160,825	36.87	86
NC_026913.1	*Q. aquifolioides* [32]	d	160,415	36.96	78
NC_029490.1	*Q. baronii* [33]	e	161,072	36.81	86
NC_031356.1	*Q. variabilis* [22]	f	161,077	36.79	86
NC_031357.1	*Q. dolicholepis* [22]	g	161,237	36.80	86
NC_036370.1	*Q. tarokoensis* [34]	h	161,355	36.80	86
NC_036930.1	*Q. glauca* [27]	i	160,798	36.90	86
NC_036936.1	*Q. tungmaiensis* [35]	j	160,702	36.94	86
NC_036941.1	*Q. sichourensis* [36]	k	160,681	36.92	86
NC_039428.1	*Q. chenii* [37]	l	161,117	36.79	86
NC_039429.1	*Q. acutissima* [37]	m	161,127	36.79	86
NC_039725.1	*Q. dentata* [38]	n	161,250	36.83	86
NC_039972.1	*Q. obovatifolia* [39]	o	160,817	36.90	86
NC_043857.1	*Q. wutaishanica* [40] *	p	161,296	36.81	85
NC_043858.1	*Q. mongolica*	q	161,194	36.83	86
NC_046388.1	*Q. robur* [41]	r	161,172	36.83	89
NC_046583.1	*Q. bawanglingensis* [24]	s	161,394	36.79	86
NC_047481.1	*Q. coccinea* [42]	t	161,298	36.80	88
NC_048488.1	*Q. phillyraeoides* [43]	u	161,384	36.79	86
NC_049876.1	*Q. gilva*	v	160,742	36.91	84
NC_050963.1	*Q. pannosa* [44]	w	161,222	36.85	85
NC_050972.1	*Q. virginiana* [45]	x	161,221	36.81	86
NC_054352.1	*Q. acuta* [46]	y	160,533	36.93	85
NC_057248.1	*Q. chungii* [47]	z	160,731	36.91	85

* This reference reported the chloroplast genome of *Q. fenchengensis* (NC_048513.1), which has the same sequence as that of *Q. wutaishanica*.

**Table 2:** Statistics of A-, C-, G- and T-terminal codon usage based on RSCU values.

Codon Bias	Ranges	A Ending *	C Ending *	G Ending *	T Ending *
Positive bias	RSCU > 1.6	32.95/23.08	3.28/0.00	2.47/0.00	40.15/56.25
	1 < RSCU ≤ 1.6	38.11/23.08	8.08/25.00	10.80/8.33	41.41/31.25
	Subtotal	71.06/46.16	11.36/25.00	13.27/8.33	81.56/87.50
Negative bias	0.6 ≤RSCU < 1	15.90/15.38	27.65/25.00	23.61/0.00	9.47/0.00
	0 < RSCU < 0.6	7.88/38.46	42.55/31.25	45.22/58.33	3.54/6.25
	Subtotal	27.50/53.84	86.49/68.75	85.65/91.66	16.04/6.25
No bias	RSCU = 1	1.43/0.00	2.15/6.25	1.08/0.00	2.40/6.25
Not in use	RSCU = 0	3.72/0.00	16.29/12.50	16.82/33.33	3.03/0.00

* In percentage number format, with genome data before the slash “/” and *psbA* gene data after the slash “/”.

**Table 3:** Test of independence between different codon positions in the 4-fold degenerate families.

Amino acids	Codons	*p* Value *	
		Genome	*psbA*
Leu/Pro/Arg	CTN/CCN/CGN	***p* < 0.001**	*p* = 0.062
Val/Ala/Gly	GTN/GCN/GGN	***p* < 0.001**	*p* = 0.026
Leu + Val/Ser + Pro + Thr + Ala/Arg + Gly	CTN + GTN/NCN/CGN + GGN	***p* < 0.001**	***p* < 0.001**
Leu/Val	CTN/GTN	***p* < 0.001**	*p* = 1.000
Arg/Gly	CGN/GGN	***p* = 0.021**	*p* = 0.597
Ser/Pro/Thr/Ala	TCN/CCN/ACN/GCN	***p* < 0.001**	*p* = 0.125

* Pearson’s Chi-square test was used for genome data and Fisher’s exact test was used for gene data. Significant *p* values after Benjamini–Hochberg correction are marked in bold (FDR = 0.05).

## Data Availability

We used genome sequences from GenBank for codon usage analysis and no new genome sequence was generated.

## Supplementary Materials

The following supporting information can be downloaded at: https://www.mdpi.com/article/10.3390/genes13112156/s1, Code S1: GC content calculation script; Code S2: Codon usage calculation script; Table S1: Nucleotide composition and codon bias in 50 genes shared by 26 *Quercus* chloroplast genomes; Table S2: Relative synonymous codon usage (RSCU) in 50 genes shared by 26 *Quercus* chloroplast genomes; Table S3: RSCU similarity and the statistical results; Table S4: Proximity matrix of mean RSCU similarity between genes; Table S5: Mean relative synonymous codon usage (RSCU) of 50 genes shared by 26 *Quercus* chloroplast genomes (*n* = 26); Table S6: Codon usage frequency statistics in all the 26 *Quercus* chloroplast genomes; Table S7. Test for context-dependent mutation in four genes.
